# Maternal synapsin autoantibodies are associated with neurodevelopmental delay

**DOI:** 10.3389/fimmu.2023.1101087

**Published:** 2023-01-19

**Authors:** Isabel Bünger, Konstantin L. Makridis, Jakob Kreye, Marc Nikolaus, Eva Sedlin, Tim Ullrich, Christian Hoffmann, Johannes Vincent Tromm, Helle Foverskov Rasmussen, Dragomir Milovanovic, Markus Höltje, Harald Prüss, Angela M. Kaindl

**Affiliations:** ^1^ Charité – Universitätsmedizin Berlin, Department of Neurology and Experimental Neurology, Berlin, Germany; ^2^ German Center for Neurodegenerative Diseases (DZNE) Berlin, Berlin, Germany; ^3^ Charité – Universitätsmedizin Berlin, Department of Pediatric Neurology, Berlin, Germany; ^4^ Charité – Universitätsmedizin Berlin, Center for Chronically Sick Children, Berlin, Germany; ^5^ Charité – Universitätsmedizin Berlin, German Epilepsy Center for Children and Adolescents, Berlin, Germany; ^6^ Charité – Universitätsmedizin Berlin, Institute of Cell- and Neurobiology, Berlin, Germany; ^7^ Berlin Institute of Health (BIH), Berlin, Germany; ^8^ Charité - Universitätsmedizin Berlin, Institute of Integrative Neuroanatomy, Berlin, Germany

**Keywords:** synapsin 1, antineuronal autoantibodies, transplacental transfer, maternofetal autoimmunity, developmental delay, epilepsy, behavioral problems

## Abstract

Maternal autoantibodies can be transmitted diaplacentally, with potentially deleterious effects on neurodevelopment. Synapsin 1 (SYN1) is a neuronal protein that is important for synaptic communication and neuronal plasticity. While monoallelic loss of function (LoF) variants in the *SYN1* gene result in X-linked intellectual disability (ID), learning disabilities, epilepsy, behavioral problems, and macrocephaly, the effect of SYN1 autoantibodies on neurodevelopment remains unclear. We recruited a clinical cohort of 208 mothers and their children with neurologic abnormalities and analyzed the role of maternal SYN1 autoantibodies. We identified seropositivity in 9.6% of mothers, and seropositivity was associated with an increased risk for ID and behavioral problems. Furthermore, children more frequently had epilepsy, macrocephaly, and developmental delay, in line with the SYN1 LoF phenotype. Whether SYN1 autoantibodies have a direct pathogenic effect on neurodevelopment or serve as biomarkers requires functional experiments.

## Introduction

Synapsin 1 (SYN1) is a neuronal phosphoprotein encoded by the *SYN1* gene and plays an important role in neurotransmitter release and neuronal plasticity (1, 2). Patients with loss of function (LoF) variants in the *SYN1* gene have been associated with X-linked phenotypes consisting of epilepsy, learning difficulties, intellectual disability (ID), macrocephaly, behavioral problems, and autism-spectrum disorders (ASD) (MIM#300491, MIM#300115) (3–5).

In recent years, antineuronal autoantibodies have been increasingly described to cause target-specific autoimmune neurologic disorders, in some cases matching the clinical phenotypes of patient carrying genetic variants of these targets. For example, both patients with variants in GABA_A_ receptor subunit genes and those with GABA_A_ receptor autoantibodies display epilepsy as a common phenotype (6–8). Autoantibodies are usually regarded as those generated within an individual; however, they can also be transferred diaplacentally from mother to child (9). As the blood brain barrier is not yet fully matured, these autoantibodies of maternal origin can target the developing brain and thereby potentially affect neurodevelopment (9). There is increasing evidence that maternal immune activation through autoantibodies can influence the occurrence of neurodevelopmental disorders, such as ASD (9). In addition, multiple murine models have demonstrated the direct deleterious effect of various antineuronal autoantibodies in offspring through gestational transfer (10–13).

In previous studies, autoantibodies against SYN1 have been identified in patients with neurologic and psychiatric disorders (14, 15). We have recently shown that SYN1 autoantibodies in pregnant women are associated with abnormalities of fetal development including intrauterine growth retardation (16). However, their role in mothers of children with defined neurologic disorders has not been studied so far. Thus, we assessed the prevalence of SYN1 autoantibodies in mothers of a cohort of 208 pediatric patients with neurological disorders.

## Material and methods

### Cell-based assay

Synapsin 1b (SYN1b) cell-based assay (CBA) was performed using human embryonic kidney (HEK293T) cells which were transfected with human SYN1b. Methanol-fixed cells were then incubated with sera diluted 1:300. IgG AF488-antibody (Dianova, #109-545-003), commercial rabbit SYN1/SYN2 antibody (Synaptic Systems, #106002) and anti-rabbit IgG AF594-antibody (Jackson IR, #111-585-003) were used to detect IgG binding. Two independent investigators scored the CBA using the following semi-quantitative score: 0, no binding; 1, unspecific signal (‘background’); 2, positive (intensive binding). Sera were tested on control cells overexpressing the NR1 subunit of the N-methyl-D-aspartate receptor (NMDAR), contactin-associated protein 2 (Caspr2), and SYN1 CBA-positive sera also on untransfected control HEK293T cells to exclude non-specific binding (14, 16).

### Enzyme-linked immunosorbent assay

For the ELISA, 200 ng of recombinant rat Syn1-His-EGFP (17) was diluted in PBS and incubated overnight at 4°C in 96-well high-binding plates. Serum (1:200 diluted in PBS containing 1% BSA, 0.05% Tween) was incubated for one hour at room temperature, on which HRP-coupled anti-human IgG antibody (Dianova, #109-035-003) was added for an additional hour. Ultra TMB substrate solution was added, and reaction terminated after one minute with H_2_SO_4_. Absorbance at 450 nm was measured and corrected with controls (mean of wells without serum and reference absorbance at 630 nm).

### Western blot

SDS-Page and Western blotting were performed as described using cortices from *Syn1/2/3* triple knockout (TKO) mice and wild type (WT) mice (14). Sera of CBA with scores of 1-2 were diluted to 1:200 and used to incubate membranes. A rabbit polyclonal SYN1/2 antibody (Synaptic Systems, #106002) was used as positive control and a mouse monoclonal antibody against glyceraldehyde-3-phosphate dehydrogenase (GAPDH, Merck Millipore, #MAB374) served as loading control.

### Clinical data and statistical analysis

We studied a cohort of 208 mothers of pediatric patients with neurological disorders treated at the Center for Chronically Sick Children, Charité – Universitätsmedizin Berlin, Germany. Healthy mothers of children with unclear developmental disabilities were approached regarding the study by a study team physician at their child’s regular treatment appointment. Due to the exploratory approach, no exclusion criteria were specified. Maternal sera were prospectively collected and analyzed. The finding of SYN1 autoantibodies did not influence the treatment of the patients. Patient data were collected retrospectively and blinded to the maternal antibody levels using a standardized data collection sheet. Statistical analysis was performed using R (RStudio version 4.2.1) and the packages *crosstable, ggplot2, UpSetR*, and *psych*. Descriptive statistics were performed to calculate percentages and frequencies. Differences in categorical variables are reported as odds ratio and 95% confidence interval. P values were calculated using the Chi-square test and Fisher’s Exact Probability Test, as applicable. Test results with p<0.05 were considered statistically significant. The study was approved by the local ethics committee (approval no. #EA2/220/20).

## Results

**Figure 1:**
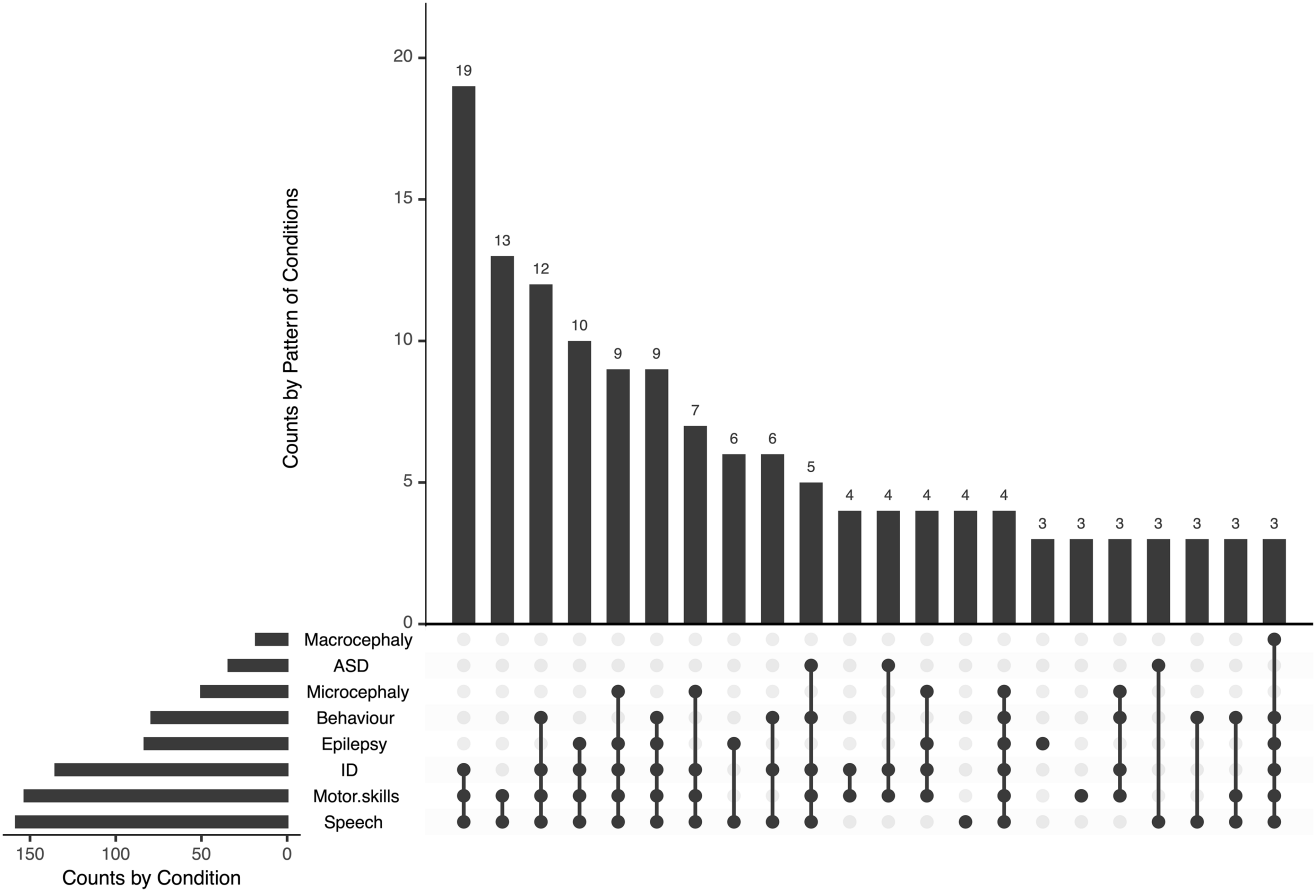
Phenotype of children of mothers tested for SYN1 autoantibodies. Developmental delay in the domains speech and motor skills were the most common findings, often associated with intellectual disability and epilepsy. ID, intellectual disability; ASD, autism-spectrum disorder.

We screened sera for SYN1 autoantibodies in 208 mothers to assess the relationship with neurodevelopment in children. Due to the explorative approach, no exclusion criteria were set. Mothers gave birth at an average age of 30.93 ± 5.81 years. Children (male n=134, 64.1%) were 6.6 ± 4.14 years old at testing. Developmental delay in the domains speech (n=158, 75.7%) and motor skills (n=153, 73.1%) were the most common findings. Furthermore, 135 patients had intellectual disability (64.4%), and 83 patients had epilepsy (39.9%) (**Figure 1**). Epilepsy (p=0.049), ASD (p=0.001) and behavioral problems (p=0.008) were more common in male, while microcephaly was more common in female patients (p=0.001).

**Figure 2:**
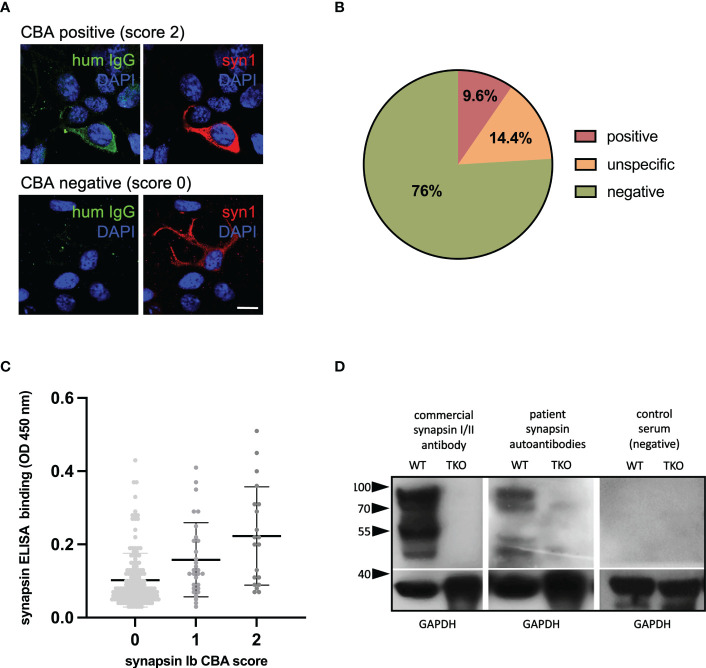
Detection of synapsin 1 (SYN1) autoantibodies. **(A)** Examples of immunofluorescence stainings, using human sera at a 1:300 dilution (green) with or without binding to HEK cells overexpressing human SYN1b are shown in the top row and bottom row. Using a commercial SYN1/2 antibody, protein expression is verified (red). DAPI is used to stain the nuclei (blue). Scale bar: 20 µm. **(B)** In 9.6% of mothers SYN1 autoantibodies were detected using a CBA. **(C)** CBA and in-house ELISA correlated weakly, with higher mean serum IgG levels in the CBA-positive group. **(D)** Representative immunoblots of wild type (WT) and Syn1/2/3 triple KO (TKO) mice cortex homogenates with a commercial synapsin 1/2 antibody as positive control, and with sera (1:200 dilution) from a CBA-positive mother and a CBA-negative control. Major bands at 80-90 kDa corresponding to the molecular weight of synapsin 1a/1b and additional bands at 50-55 kDa that could represent the synapsin 2b isoform or breakdown products of synapsin 1 were detected in wild type but not in TKO mouse tissue by both the commercial antibody and the serum of the CBA-positive mother. Detection of GAPDH served as loading control. HEK human embryonic kidney; CBA, cell-based assay; ELISA, enzyme-linked immunosorbent assay, WB, western blot.

In a first step, the patients’ mothers were screened using the previously described CBA (14). We used a visual scoring system for antibody binding in the CBA, and scores of 2 (intensive binding) were considered as positive (**Figure 2A**). Positive binding was identified in 20 mothers (9.6%). In 30 mothers, unspecific binding was detected while all others (n=158) showed no binding (**Figure 2B**). We further analyzed the sera of mothers with a SYN1 ELISA. Here, we found only a weak correlation between the CBA and ELISA results (r=0.29, **Figure 2C**). Possibly, these differing results can be explained by the binding of SYN1 autoantibodies to conformational epitopes that are not detected by ELISA. Therefore, we performed Western blots using the sera and cortex homogenates of wild-type and *SynI/II/III* triple knockout mice. We detected IgG binding to wild-type mouse brain homogenates in four of the 20 CBA-positive sera (**Figure 2D**). The other sera did not show immunoreactive bands at the expected molecular weight. These results point towards SYN1 IgG autoantibodies binding to linear SYN1 epitopes in only a small group, whereas autoantibodies against conformational synapsin epitopes predominate.

We further analyzed children of mothers with a positive CBA (**Table 1**). Most commonly the children had ID (n=18, 90%), speech delay (n=17, 85%), motor delay (n=17, 85%), and epilepsy (n=12, 60%). Seven patients had a developmental and epileptic encephalopathy (DEE). Cranial MRI was performed in 15 patients, with abnormalities detected in eight patients (53.3%, **Table 1**). Genetic testing was carried out in 13 patients, with variants (n=7) and microdeletions (n=1) identified in eight patients (61.5%). Of these seven variants, one was classified as pathogenic (14.3%), three as probably pathogenic (42.9%), and three as variants of unclear significance (42.9%). Identified variants (gene, cDNA, ACMG classification and NM number) are listed in **Table 1** for each patient. The four patients with IgG binding to wild-type mouse brain, showed no striking phenotypic differences to patients without binding (**Table 1**).

**Table 1:** Phenotype of patients with positive maternal synapsin I (SYN1) autoantibodies.

no.	sex	age at test (years)	ID	cMRI	Epilepsy; Seizures	Gene name	cDNA position	ACMG	NM number	OMIM	Speech	Motor skills	ASD	Behavior	MIC	MAC
2	m	7.84	+	CM	DEE; focal-onset atonic seizures	*AIFM1*	c.1465G>A	III	004208.3	300169	+	+	–	+	–	+
7	m	2.47	+	HC	DEE; generalized tonic-clonic seizures	1,2,3					+	+	–	+	–	+
22	m	4.24	+	n.p.	–	1,2					+	+	+	+	–	–
26	m	0.72	+	PCH	DEE; epileptic spasms	n.p.					+	+	–	+	+	–
41	m	1.02	–	peritrigonal localized dysmyelination	–	*PTEN*	c.441A>T	IV	000314	601728	+	+	–	–	–	+
42	f	0.34	+	lissencephaly	DEE; focal onset clonic seizures without awareness	microdel. 17p13.3				247200, MDLS	+	+	–	–	–	–
46	m	1.26	+	delayed myelination	DEE; epileptic spasms	*1,2,3*				617057	+	+	+	–	+	–
60	m	4.59	+	NAD	focal onset seizures without awareness	n.p.					+	+	–	+	–	–
71	f	13.77	+	NAD	generalized tonic-clonic seizures	1,2					+	–	–	+	–	–
72	m	10.07	+	NAD	DEE; focal onset seizures without awareness	n.p.					+	+	–	+	+	–
86	m	5.06	+	NAD	–	*DPYD*	c.623G>T	III	000110	612779	+	+	+	+	–	–
94	m	3.92	+	NAD	–	n.p.					–	+	+	–	–	–
106	f	12.28	+	n.p.	–	*ANO5*	c.172C>T	IV	213599.2	608662	+	+	–	+	–	+
146	f	15.68	+	infra- and supratentorial atrophy	DEE; focal onset myoclonic seizures without awareness	*SCN1A*	c.3887T>C	III	001165963	182389, DS	+	+	–	–	+	–
152	m	5.06	+	n.p.	–	*DDB1*	c.637G>A	IV	001923	600045	+	+	–	–	–	–
158	m	4.06	+	n.p.	–	n.p.					+	+	–	+	–	–
159	f	3.97	+	SBH	focal onset myoclonic seizures without awareness	*DCX*	c.814C’T	V	000555	300121	+	+	–	–	+	–
161	m	4.62	+	n.p.	–	1					+	+	–	+	–	–
168	m	16.62	+	NAD	generalized tonic-clonic seizures	n.p.					–	–	–	+	+	–
208	m	6.94	–	NAD	epilepsy of unknow cause with tonic clonic seizures	n.p.					–	–	–	–	–	–

ASD, autism-spectrum disorder; CM, Chiari malformation; DEE, Developmental and epileptic encephalopathy; DS, Dravet syndrome; HC, Hydrocephalus communicans; ID, intellectual disability; MAC, macrocephaly; MIC, microcephaly; MDLS, Miller-Dieker-Lissencephaly syndrome; NAD, no abnormality detected; n.p., not performed; PCH, Pontocerebellar hypoplasia; SBH, subcortical band heterotopia; Genetic analysis: 1, chromosome analysis; 2, microarray-based comparative genomic hybridization (Array-CGH); 3, trio whole-exome analysis.

**Figure 3:**
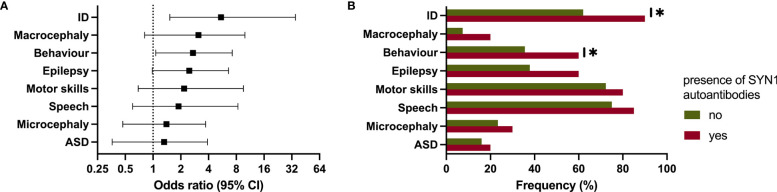
Clinical phenotype in patients of mothers with autoantibodies against synapsin 1 (SYN1). **(A, B)** Forest plot and bar chart showing the significantly increased risk for intellectual disability and behavioral problems. ID, intellectual disability; ASD, autism-spectrum disorder. * p ≤ 0.05.

We next analyzed the clinical data based on the results of the CBA. While there was no association between CBA and age of mothers, age at birth or children’s age, we found an increased risk for specific phenotypes in patients of mothers with positive CBA. A positive CBA was a significant risk factor in their children for the presence of ID (OR: 5.46 [95% CI: 1.52 - 35.01], p = .0134) and behavioral problems (2.71 [1.07 - 7.23], p = .0328). Furthermore, patients showed a tendency for the presence of macrocephaly (3.11 [0.81 - 9.92], p = .0785), epilepsy (2.47 [0.98 - 6.59], p = .0536), ASD (1.32 [0.36 - 3.89], p = .7493), microcephaly (1.40 [0.47 - 3.72], p = .5882), and a developmental delay in the domains speech (1.89 [0.60 - 8.34], p = .4167) and motor skills (2.17 [0.69 - 9.55], p = .2223) (**Figures 3A, B**).

## Discussion

In this study, we recruited a clinical cohort of 208 mothers and their children and analyzed the prevalence of maternal SYN1 autoantibodies and their association with developmental phenotypes. SYN1 IgG autoantibodies were detected through CBA in 9.6% of mothers with neurologically sick children. Most of the SYN1 CBA positive cases were negative on Western blots under denaturing conditions, suggesting a conformational dependency of their target binding, as similarly observed in other human antineuronal autoantibodies (18). In a study by Höltje et al. that analyzed SYN1 autoantibodies in a cohort of patients with various psychiatric and neurological disorders, binding was detected in immunoblots in about half of all patients (14). It is unclear whether in less denaturing conditions more SYN1 CBA positive cases would be positive. As in this study, we recently reported IgG binding in immunoblots of 263 pregnant women screened for synapsin I autoantibodies in only 22.9% and found no correlation between ELISA and CBA antibody levels (16).

SYN1 is member of the family of synapsins, encoded by the three genes *SYN1, SYN2*, and *SYN3* in humans. SYN1 regulates the trafficking of synaptic vessels between the readily releasable pool and reserve pool. Furthermore, Syn1 moderates neuronal development through neurite outgrowth and synaptogenesis (2). *Syn1* mutant mice have severe epilepsy with generalized seizures (19).

In this present study of 208 children with various neurologic disorders, a positive maternal CBA showed a significant association with ID and behavioral problems, while there was and a trend towards other abnormalities such as macrocephaly and epilepsy. This is intriguing given that patients with LoF variants in *SYN1* display a similar phenotype with epilepsy, ID, ASD, macrocephaly, and behavioral abnormalities (3–5). The lack of a clear association to these phenotypes may relate to the low statistical power of the study and emphasizes to investigate larger cohorts.

When further analyzing the 20 CBA-positive patients, seven had identified genetic variants/microdeletions of which one was considered pathogenic and three as likely pathogenic variants. This raises speculation as to whether genetic alterations in brain proteins may lead to neoantigen formation and increased autoimmunity. Autoantibodies arise from multilayered defects at immune checkpoints due to dysregulated negative selection of B cells which eventually develop into plasma cells or B memory cells (18). These autoantibodies can act through target-specific mechanisms. For example, *in vitro* experiments could show that SYN1 autoantibodies can be internalized through FcγII/III-mediated endocytosis and promote SYN1 aggregation resulting in impairment of synaptic transmission (20). Future experimental models will have to elucidate the functional role of maternal SYN1 autoantibodies and investigate whether they can reach the fetal brain and have a direct pathogenic effect on fetal development *in vivo* or rather represent a clinical biomarker of neuronal cell damage. Synaptic impairment by maternal SYN1 autoantibodies with possible detrimental effects on neurodevelopment may have a similar effect as other maternal antineuronal autoantibodies such as NMDAR, Caspr2, and aquaporin-4 antibodies, for which experimental studies have demonstrated pathogenic effects (10–13). Further, Caspr2 autoantibodies present during pregnancy were associated with ID and developmental disorders (21). However, functional studies on the effects of SYN1 autoantibodies on neurodevelopment are lacking and should be performed using patient-derived monoclonal antibodies (22).

In addition, it is unclear to what extent SYN1 autoantibodies cross the placenta and can be found in CSF. For this, functional experiments are necessary, in which human monoclonal autoantibodies are systemically applied into pregnant mice. It would be further interesting to assess if children of mothers with a positive CBA have SYN1 autoantibodies also in their CSF.

The study has multiple limitations including heterogeneity of the pediatric cohort. Other environmental factors, besides identified genetic variants, can also have an influence on the phenotype of these children. Furthermore, mothers were tested after pregnancy, Thus, it is unclear whether they were also positive during pregnancy which does not allow a direct statement about causality. To assess whether SYN1 autoantibodies have an effect during pregnancy we recently reported on a cohort of 263 pregnant women with seropositivity for SYN1 autoantibodies in 13.3%, matching the prevalence in this cohort (16). Seropositivity was associated with abnormalities of fetal development including intrauterine growth retardation, structural abnormalities and amniotic fluid disorders. The next step is to accurately phenotype these patients to assess the impact of SYN1 during pregnancy on the neurodevelopment of these children. Furthermore, the results of this study need to be validated by larger prospective multicentric studies with age-matched controls of healthy patients to increase power and counteract selection bias.

Despite these limitations, the study indicates a potential influence of maternal SYN1 autoantibodies on neurodevelopment. These results do not currently trigger any direct therapeutic or diagnostic consequences, but could influence standard treatment of pregnant women in the long term and possibly lead to new therapeutic approaches.

## Data availability statement

The original contributions presented in the study are included in the article/supplementary materials. Further inquiries can be directed to the corresponding author.

## Ethics statement

The studies involving human participants were reviewed and approved by Charité - Universitätsmedizin Berlin (approval no. #EA2/220/20). Written informed consent to participate in this study was provided by the participants’ legal guardian/next of kin. The animal study was reviewed and approved by Charité - Universitätsmedizin Berlin (approval no. #EA2/220/20).

## Author contributions

Conceptualization: AK and HP. Patient recruitment: IB, KM, TU and ES. CBA and ELISA testing: IB, JK, CH, HR, MH, DM and JT. Western blotting: MH. Data collection, analysis, and visualization: IB and KM. Resources: AK and HP. Writing – original draft: IB, KM. Writing – review & editing: all authors. All authors contributed to the article and approved the submitted version.
